# Vertebral osteomyelitis with *Campylobacter jejuni* – a case report and review of the literature of a very rare disease

**DOI:** 10.5194/jbji-9-59-2024

**Published:** 2024-02-12

**Authors:** Simone Greminger, Carol Strahm, Julia Notter, Benjamin Martens, Seth Florian Helfenstein, Jürgen Den Hollander, Manuel Frischknecht

**Affiliations:** 1 Medical Center Pflanzberg, Poststrasse 20, 8274 Tägerwilen, Switzerland; 2 Division of Infectious Diseases, Cantonal Hospital St Gallen, Rorschacher Strasse 95, 9007 St Gallen, Switzerland; 3 Center for Spine Surgery Eastern Switzerland, Cantonal Hospital St Gallen, Rorschacher Strasse 95, 9007 St Gallen, Switzerland; 4 Division of General Internal Medicine, Cantonal Hospital St Gallen, Rorschacher Strasse 95, 9007 St Gallen, Switzerland; 5 Cantonal network of Radiology and Nuclear Medicine, Cantonal Hospital St Gallen, Rorschacher Strasse 95, 9007 St Gallen, Switzerland

## Abstract

Infections with *Campylobacter* species mainly cause gastrointestinal disease and are usually self-limiting. Systemic complications such as bacteremia and osteoarticular infections are rare. Here we report a very rare case of a vertebral osteomyelitis due to *C. jejuni*, and we reviewed the literature for similar cases, identifying six other cases. Therapy should be guided on resistance testing if available due to emerging resistance rates, especially to fluoroquinolones. Azithromycin may be a treatment option for *C. jejuni* spondylodiscitis.

**Figure 1:**
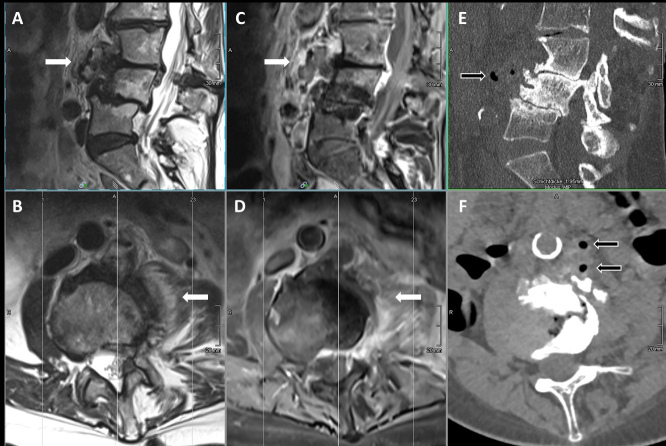
MRI (T2w images sagittal **(a)** and axial **(b)**; T1w + gadolinium images sagittal **(c)** and axial **(d)**); and CT-scan images (sagittal in bone window **(e)** and axial in soft tissue window **(f)**) of the lumbosacral spine before treatment, showing marked degenerative changes in the lower lumbar spine but additionally pronounced contrast enhancement in the paravertebral tissue and psoas muscle (white arrows) with gas containing small abscesses prevertebral (black arrows) hinting to a masked pyogenic spondylodiscitis with little involvement of the intervertebral space.

## 1 Case report

A 77-year-old female patient suffering from chronic lower-back pain due to scoliosis with multiple degenerative changes and discus herniation L1-S1 (last spinal infiltration more than 10 months ago) presented to a local hospital because of severe persisting diarrhea for about 7 d and acute exacerbation of lower-back pain for 2 d. The patient was under daily steroids (prednisone 10 mg) because of a rheumatic polymyalgia and suffered from secondary osteoporosis and restless legs. Their vital signs, including body temperature, were normal. Laboratory investigation revealed an inflammatory status with elevated C-reactive protein (CRP) of 376 mg L^-1^ (normal <8 mg L^-1^), elevated total white blood cell count of 17.9 G L^-1^ (normal range 4.0–10.0 G L^-1^) without left shift, thrombocytosis of 430 G L^-1^ (normal range 150–300 G L^-1^), and mild hyponatremia. Liver and kidney function were normal. Except for the pre-existing sensitivity loss in the dermatome L4 on the left side and slight abdominal tenderness, no focal neurologic deficits or further hints for other sites of infection could be found. A magnetic resonance imaging (MRI) of the lumbosacral spine showed inflammatory changes paravertebrally and inside the psoas muscle on the left side at level L3/4 without clear involvement of the intervertebral disc compartment yet, hinting at a (because of marked degenerative changes) masked spondylodiscitis with little involvement of the intervertebral space (Fig. 1). A small ( 1.5×1.5 cm), not drainable, abscess formation was confirmed by CT scan, revealing gas formation in the soft tissues. Two days after admission, *Campylobacter jejuni* could be detected in aerobic blood and stool cultures. An antimicrobial treatment with azithromycin 500 mg once daily (initially intravenously) was initiated. Antibiotic susceptibility testing showed the following minimal inhibitory concentrations (MICs): ciprofloxacin MIC 0.125 $µg\;{\mathrm{mL}}^{-1}$ , erythromycin MIC 2.0 $µg\;{\mathrm{mL}}^{-1}$ , imipenem MIC 0.125 $µg\;{\mathrm{mL}}^{-1}$ , gentamicin MIC 0.75 $µg\;{\mathrm{mL}}^{-1}$ , and amoxicillin MIC 3.0 $µg\;{\mathrm{mL}}^{-1}$ . According to the European Committee on Antimicrobial Susceptibility Testing (EUCAST) criteria, this is considered susceptible to ciprofloxacin under increased drug exposure ( = I) and susceptible to erythromycin under standard dosing regimen ( = S) according to the EUCAST definitions of susceptibility testing categories from 2019. At the time of presentation, the former EUCAST criteria interpreted the above MICs as susceptible ( = S) (EUCAST, 2023). A CT-guided fine-needle biopsy of the psoas muscle was performed: bacterial cultures showed no growth, but 16S rRNA polymerase chain reaction (PCR) analysis turned out to be positive for *Campylobacter* species. The antibiotic treatment was switched to oral ciprofloxacin 750 mg twice a day.

**Figure 2:**
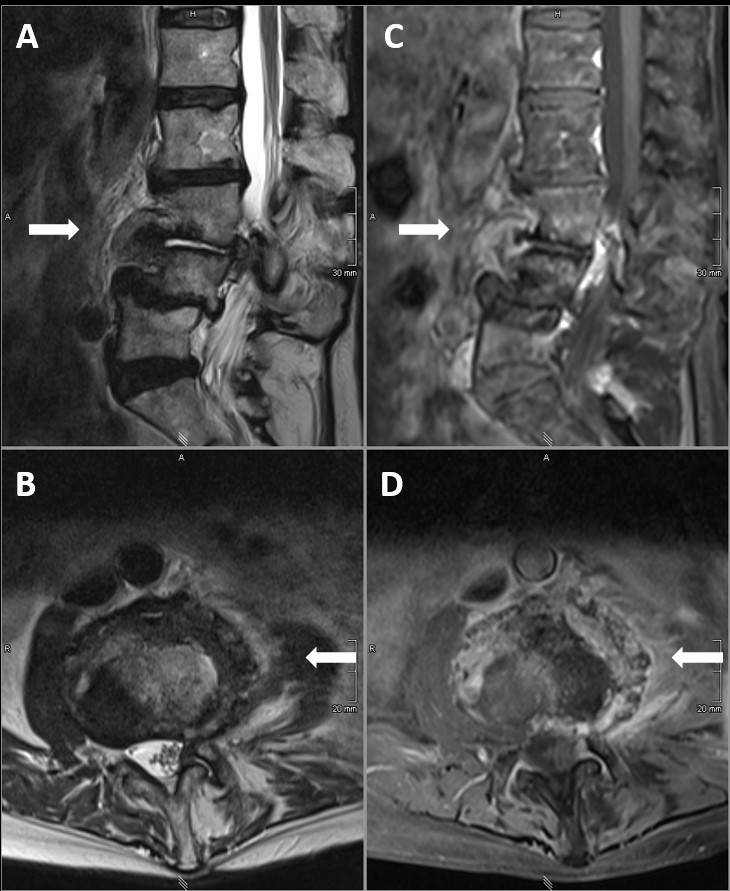
MRI (T2w images sagittal **(a)** and axial **(b)**; T1w + gadolinium images sagittal **(c)** and axial **(d)**) of the lumbosacral spine after treatment, showing marked improvement of the infectious changes in the prevertebral and paravertebral tissue (white arrows).

Under therapy, the diarrhea stopped quickly and inflammatory markers turned to normal range within a few weeks. Due to a significant rise of liver enzymes 23 d after initiation of treatment (ASAT/ALATASAT is aspartate aminotransferase, ALAT is alanine aminotransferase, and GGT is gamma-glutamyltransferase.

>2 times the upper limit, GGT >30 times the upper limit), presumably drug-induced by ciprofloxacin, azithromycin was reinitiated with consequently rapid normalization of liver parameters, so we refrained from further examination of the hepatopathy.

The patient was treated for a total of 10 weeks. MR imaging at the end of therapy showed a significant decrease in inflammatory reaction and complete resolution of the abscess formations in the psoas muscle. However, the imaging also revealed clear involvement of intervertebral disc and the adjacent vertebral bodies, so spondylodiscitis was retrospectively proven (Fig. 2). The lower-back pain also improved but did not resolve completely because of the patient's pre-existing chronic lumbar back pain syndrome. No clue regarding the environmental source of infection of the *Campylobacter jejuni* could be found in the patient's medical history.

## 2 Literature review for *Campylobacter jejuni* spondylodiscitis

We screened the PubMed database using the following keywords: “Campylobacter” AND “spondylodiscitis”; “Campylobacter” AND “vertebral osteomyelitis”; and “Campylobacter” AND “bacteremia”. A total of six cases published from 2011 to 2022 were identified. They are described in detail in Tables 1 and 2 (Feodoroff et al., 2011; Ajili et al., 2011; Tappe et al., 2012; Puljiz and Topic, 2017; Langereis et al., 2018; Nakatani et al., 2022). Most patients were elderly and had underlying comorbidities like diabetes, liver cirrhosis, or a history of chronic back disorders. If the location of the spondylodiscitis was reported, it was mostly the lumbar spine that was affected. Only half of the patients suffered from diarrhea and/or fever. Four out of six were bacteremic at the time of presentation. Two articles reported susceptibility to ciprofloxacin. The others did not list detailed data on antibiotic susceptibility. Antimicrobial treatment varied. However, most cases were treated with either fluoroquinolones or macrolides. A total treatment duration between 4–36 weeks (median 8 weeks) was reported. A favourable outcome was described in all cases but with only scarce descriptions of follow-up time.

**Table 1:** Identified cases of *Campylobacter jejuni* spondylodiscitis in literature review and our case: clinical features.

Case	Study	Sex and age (years)	Comorbidities	Infect. location	Symptoms	Pathologic laboratory results	Suspected source ofinfection
1	Feodoroff et al. (2011)	f, 46	None	No data	Diarrhoea, fever, back pain	No data	No data
2	Ajili et al. (2011)	m, 34	Heavy smoker, discal hernia	L4/5	Fever, back pain	CRP 49 mg L^-1^,WBC 8 G L^-1^	No data
3	Tappe et al. (2012)	f, 68	Diabetes with polyneuropathy, hypertension, restless legs syndrome, hyperthyroidism, coxarthrosis, cervicobrachialgia	L2/3 Abscess leftpsoas muscle	Back pain	CRP 90 mg L^-1^, WBC 20.4 G L^-1^,Tc 572 G L^-1^	No data
4	Puljiz and Topic (2017)	m, 59	Degenerative lower-back pain, Meniere's disease	L4/5 Epidural abscess	Diarrhoea, fever, back pain	CRP 77 mg L^-1^,WBC 11 G L^-1^	No data
5	Langereis et al. (2018)	m, 16	Agammaglobulinemia	L4/5	Lower-backpain	CRP 37 mg L^-1^,WBC 12 G L^-1^	No data
6	Nakatani et al. (2022)	m, 56	Dilated cardiomyopathy, ventricular tachycardia (with implantable cardioverter defibrillator (ICD)), alcohol-related liver cirrhosis	C3–C4	Neck pain	CRP 12 mg L^-1^,WBC 8.7 G L^-1^	Pork liver
7	Our case	f, 77	Osteoporosis, scoliosis, discus hernia, rheumatic polymyalgia, restless legs	L3/4 Abscess leftpsoas muscle	Diarrhoea, back pain	CRP 376 mg L^-1^,WBC 17.9 G L^-1^,Tc 430 G L^-1^	No source identified

Abbreviations: f: female, m: male, WBC: total white blood cell count, Tc: thrombocyte, CRP: C-reactive protein, G L^-1^: giga per litre.

**Table 2:** Identified cases of *Campylobacter jejuni* spondylodiscitis in literature review and our case: microbiological features, treatment and outcome.

Case	Biopsy	Bacteremia	Susceptibility testing	Stool sample positive	Antibiotic treatment	Duration treatment (total, weeks)	Surgical treatment	Time of follow-up (weeks)	Outcome
1	Culture negative	No data; but probably yes (MICs available)	Erythromycin (MIC 2 $µg\;{\mathrm{mL}}^{-1}$ )	No data	Roxithromycin	8	No data	No data	No data
2	Culture positive	No	No data	No data	Ofloxacin 200 mg bid and clindamycin 600 mg tid	12	No	No data	Favourable
3	Culture negative 16s rRNA PCR: *C. jejuni/coli*; specific PCR: *C. jejuni*	No	No	No	Ciprofloxacin 400 mg bid (iv for 1 week) and meropenem 1 g tid iv	4	No	Lost to follow-up	No data
4	None	Yes	No data	Yes	Ciprofloxacin 400 mg bid (iv for 4 weeks), 500 mg bid po	6	No	6	Favourable
5	None	Yes	Tetracycline (MIC 8 $µg\;{\mathrm{mL}}^{-1}$ ), erythromycin/azithromycin (MIC 4 $µg\;{\mathrm{mL}}^{-1}$ ), ciprofloxacin (MIC 0.125 $µg\;{\mathrm{mL}}^{-1}$ ), amoxicillin (MIC 256 mg L^-1^), amoxicillin-clavulanic acid (MIC 2 $µg\;{\mathrm{mL}}^{-1}$ )	No data	Azithromycin 500 mg qd po	36	No	36	Favourable
6	None	Yes	Ciprofloxacin susceptible (no MIC)	No data	Azithromycin 500 mg, then ciprofloxacin 400 mg bid iv 4 weeks, then ciprofloxacin 500 mg bid po	8	No	No data	Favourable
7	Culture negative 16s rRNA PCR: *Campylobacter* species	Yes (*C. jejuni*)	Ciprofloxacin (MIC 0.125 $µg\;{\mathrm{mL}}^{-1}$ ), erythromycin (MIC 2.0 $µg\;{\mathrm{mL}}^{-1}$ ), imipenem (MIC 0.125 $µg\;{\mathrm{mL}}^{-1}$ ), gentamicin (MIC 0.75 $µg\;{\mathrm{mL}}^{-1}$ ), amoxicillin (MIC 3.0 $µg\;{\mathrm{mL}}^{-1}$ )	Yes	Ciprofloxacin 750 mg bid po, then azithromycin 500 mg qd po	10	No	220 (4 years)	Favourable

Abbreviations: MIC: minimal inhibitory concentration, qd: once a day, bid: twice a day, tid: three times a day, iv: intravenous, po: per os

## 3 Discussion

*Campylobacter* is a genus of gram-negative curved or rode-shaped bacteria and is found in a wide range of animals: poultry (mainly *C. jejuni* but also *C. coli*), pigs (mainly *C. coli*), and also in pets like dogs and cats (Keller et al., 2007). Transmission to humans occurs by handling or consumption of raw, poorly cooked, or cross-contaminated food products of these animals (mainly poultry); unpasteurized cow milk; contaminated water; or by direct contact with animals (Hansson et al., 2018).

Infections in humans are mainly due to *C. jejuni* or *C. coli* (more than 90 % of all cases) and *C. fetus* (Hansson et al., 2018) and present as a gastrointestinal disease with mild to severe diarrhea, abdominal cramping, nausea, fever, and sometimes bloody diarrhea. In developed countries *Campylobacter* enteritis is the major cause of food-borne bacterial enteritis, mainly caused by *C. jejuni* or *C. coli* (Hansson et al., 2018). Incubation time varies from 1 to 7 d (mean 3 d) (Hansson et al., 2018). In immunocompetent patients, *Campylobacter* enteritis is usually a self-limiting disease that resolves within 1 week with no need of antibiotic treatment (Hansson et al., 2018). Antimicrobial therapy reduces duration of intestinal symptoms only by approximately 1 d (Ternhag et al., 2007). Treatment should be considered for patients with severe symptoms or those at risk of severe disease (immunocompromised, elderly, or pregnant populations) or when bacteremia is present (Hansson et al., 2018).

*Campylobacter* bacteremia is still considered a rare event (1 % of *Campylobacter* infections) (Tinévez et al., 2022) with an incidence between 0.20–1.0 cases per 100 000 population in high-income settings (Moffatt et al., 2021). Recent data showed that *C. jejuni* is now the most frequently detected species in patients with bacteremia in a retrospective French study (Ternhag et al., 2007). Most patients with bacteremia are elderly and have underlying medical conditions (immunodepression, hematologic malignancies, solid neoplasm, diabetes) (Tinévez et al., 2022). Cases of osteoarthritis, meningitis, intra-abdominal infection (like cholecystitis, peritonitis, colitis), endocarditis, or peri-/myocarditis are described (Tinévez et al., 2022). Rare late-onset complications of *Campylobacter* infection are reactive arthritis, Guillain-Barré syndrome, and irritable bowel disease (Feodoroff et al., 2011; Hansson et al., 2018).

Vertebral osteomyelitis or spondylodiscitis by *C. jejuni* seems to be very rare. To the best of our knowledge, there are only six cases published so far. We now describe a further case consistent with the other cases: our patient is elderly, has comorbidities, is immunocompromised, and presented with febrile diarrhea and back pain as the main symptoms. Given the constellation of MRI findings, positive blood cultures, and PCR in CT-guided biopsy of psoas abscess, we were able to prove spondylodiscitis due to *Campylobacter* species infection. We did not perform an additional species-specific PCR analysis because clinical findings with proven bacteremia of *C. jejuni* were consistent with hematogenous spondylodiscitis.

In general, *Campylobacter* remains susceptible to many antibiotics, but resistance is emerging, especially to fluoroquinolones: resistance rates exceed 80 % in Southeast Asia (Mason et al., 2017), but current data from the European Centre for Disease Prevention and Control (ECDC) from 2020–2021 also show high ciprofloxacin resistance rates with an average level of 64.5 % and 69.6 % (*C. jejuni* and *C. coli,* respectively) in European countries (EFSA, 2023). Intrinsic resistance to many beta-lactam (e.g. penicillin) or cephalosporin and trimethoprim antibiotics are known (Lariviere et al., 1986). Standard therapy consists of macrolides (azithromycin); fluoroquinolones (if susceptible); or amoxicillin/clavulanic acid, carbapenems, or aminoglycosides in severely ill patients who cannot tolerate oral therapy (Dai et al., 2020).

In contrast to most other published cases of vertebral osteomyelitis, we were able to perform susceptibility testing. We preferred ciprofloxacin over azithromycin due to good bioavailability, bone penetration, and broad experience in osteomyelitis treatment (Thabit et al., 2019; Berbari et al., 2015), but we nevertheless had to switch to the macrolide because of a suspected drug-induced cholestatic hepatopathy. Azithromycin bone penetration seems to be high (Landersdorfer, 2009), but it is not used in standard treatment regimens in bone and joint infections (e.g. in staphylococci; Kim et al., 2014) with the exception of atypical mycobacteria, where macrolides are often the backbone therapy in combination with other active substances (Griffith et al., 2007). Azithromycin has successfully been used as a step-down oral therapy or in combination with other antibiotics in treatment of osteomyelitis with rare or resistant pathogens like *Salmonella typhi* (Ayeni and Calver, 2012), *Bartonella henselae* (Aparicio-Casares et al., 2021), or resistant pneumococci (Riordan et al., 2008), and it may be an option for treatment in combination with rifampicin (Shirtliff et al., 1999; O'Reilly et al., 1992). Although good quality evidence is still lacking, azithromycin may be a treatment option in select cases.

Optimal treatment duration of vertebral osteomyelitis is debated, with some proposing 6 weeks (Berbari et al., 2015; Bernard et al., 2015) and others advocating a minimal duration of 8 weeks in high-risk patients (including undrained paravertebral/psoas abscesses) (Park et al., 2016). While optimal treatment duration for *C. jejuni* spondylodiscitis is not known, most reported cases had a favourable outcome after 4–8 weeks of therapy. Our patient was treated for a total of 10 weeks with good clinical outcome and complete resolution of the abscess in MR imaging.

## 4 Conclusion

In conclusion, we report a successful treatment of a rare spondylodiscitis and concomitant psoas abscess with *C. jejuni* with a follow-up over 4 years after completion of treatment. Although clinical data is scarce, azithromycin may occasionally be a valuable treatment option for bone infections due to its good bone penetration.

## Data Availability

No data sets were used in this article.
